# Investigating Protective and Risk Factors and Predictive Insights for Aboriginal Perinatal Mental Health: Explainable Artificial Intelligence Approach

**DOI:** 10.2196/68030

**Published:** 2025-04-30

**Authors:** Guanjin Wang, Hachem Bennamoun, Wai Hang Kwok, Jenny Paola Ortega Quimbayo, Bridgette Kelly, Trish Ratajczak, Rhonda Marriott, Roz Walker, Jayne Kotz

**Affiliations:** 1 School of Information Technology Murdoch University Perth Australia; 2 Ngangk Yira Institute for Change Murdoch University Perth Australia; 3 School of Nursing and Midwifery Edith Cowan University Perth Australia

**Keywords:** explainable AI, perinatal mental health, AI-assisted decision-making, perinatal, mental health, artificial intelligence, predictive, depression, anxiety, maternal health, maternal, infant health, infant, Aboriginal, woman, psychological risk, mother, decision-making, decision support, machine learning, psychological distress, Aboriginal mothers, risk factors, Australia, cultural strengths, protective factors, life events, worries, relationships, childhood experiences, domestic violence, substance use

## Abstract

**Background:**

Perinatal depression and anxiety significantly impact maternal and infant health, potentially leading to severe outcomes like preterm birth and suicide. Aboriginal women, despite their resilience, face elevated risks due to the long-term effects of colonization and cultural disruption. The Baby Coming You Ready (BCYR) model of care, centered on a digitized, holistic, strengths-based assessment, was co-designed to address these challenges. The successful BCYR pilot demonstrated its ability to replace traditional risk-based screens. However, some health professionals still overrely on psychological risk scores, often overlooking the contextual circumstances of Aboriginal mothers, their cultural strengths, and mitigating protective factors. This highlights the need for new tools to improve clinical decision-making.

**Objective:**

We explored different explainable artificial intelligence (XAI)–powered machine learning techniques for developing culturally informed, strengths-based predictive modeling of perinatal psychological distress among Aboriginal mothers. The model identifies and evaluates influential protective and risk factors while offering transparent explanations for AI-driven decisions.

**Methods:**

We used deidentified data from 293 Aboriginal mothers who participated in the BCYR program between September 2021 and June 2023 at 6 health care services in Perth and regional Western Australia. The original dataset includes variables spanning cultural strengths, protective factors, life events, worries, relationships, childhood experiences, family and domestic violence, and substance use. After applying feature selection and expert input, 20 variables were chosen as predictors. The Kessler-5 scale was used as an indicator of perinatal psychological distress. Several machine learning models, including random forest (RF), CatBoost (CB), light gradient-boosting machine (LightGBM), extreme gradient boosting (XGBoost), k-nearest neighbor (KNN), support vector machine (SVM), and explainable boosting machine (EBM), were developed and compared for predictive performance. To make the black-box model interpretable, post hoc explanation techniques including Shapley additive explanations and local interpretable model-agnostic explanations were applied.

**Results:**

The EBM outperformed other models (accuracy=0.849, 95% CI 0.8170-0.8814; *F*_1_-score=0.771, 95% CI 0.7169-0.8245; area under the curve=0.821, 95% CI 0.7829-0.8593) followed by RF (accuracy=0.829, 95% CI 0.7960-0.8617; *F*_1_-score=0.736, 95% CI 0.6859-0.7851; area under the curve=0.795, 95% CI 0.7581-0.8318). Explanations from EBM, Shapley additive explanations, and local interpretable model-agnostic explanations identified consistent patterns of key influential factors, including questions related to “Feeling Lonely,” “Blaming Herself,” “Makes Family Proud,” “Life Not Worth Living,” and “Managing Day-to-Day.” At the individual level, where responses are highly personal, these XAI techniques provided case-specific insights through visual representations, distinguishing between protective and risk factors and illustrating their impact on predictions.

**Conclusions:**

This study shows the potential of XAI-driven models to predict psychological distress in Aboriginal mothers and provide clear, human-interpretable explanations of how important factors interact and influence outcomes. These models may help health professionals make more informed, non-biased decisions in Aboriginal perinatal mental health screenings.

## Introduction

Perinatal depression and anxiety (PNDA) negatively impact the health and well-being of mothers and babies, and disrupt maternal /infant bonding [1]. Recent studies highlighted the significant association between PNDA and adverse outcomes, including suicidal behaviors and self-harm thoughts during and after pregnancy. Roddy Mitchell et al [2] emphasized the increased risk of preterm birth, stillbirth, and suicide associated with PNDA. Furthermore, Hummel et al [3] highlighted the association of PNDA with adverse infant outcomes such as preterm birth, intrauterine growth restriction, and low birth weight. The loss of an infant's mother through suicide profoundly impacts the infant’s social and emotional well-being [4]. Many Aboriginal women experience strong social and emotional well-being and have flourishing infants and families. However, at a population level, too many Aboriginal women face the increased risk of triggering or worsening depression/anxiety directly resulting from the enduring challenges, barriers, and adversities from colonization, cultural disruption, and past and present policies such as the Stolen Generations. These interrelated risks include poverty, racism, intergenerational and complex trauma, racism, cultural bias, loss of cultural identity, and other inequities [5,6]. A systematic review by Owais et al [7] indicated Aboriginal women face a 38% higher chance of experiencing depression, are 79% more susceptible to mental-health problems during pregnancy, and are 30% more likely to endure mental health complexities post giving birth. A Western Australian study revealed that between 1997 and 2013, one in 3 Aboriginal babies were born to mothers who sought hospital care for mental health issues related to substance abuse, depression, or anxiety [8]. Despite routine screening for PNDA and anxiety in Australia for over 20 years, the gap in Aboriginal mothers’ and infants’ health and well-being remains unacceptable across all key indicators. This is evident in disproportionately higher rates of premature births, low birth weight babies, and child removal [5,6]. Conventional health systems’ approach to antenatal/postnatal care and screening are often culturally insensitive and retraumatizing for Aboriginal women [9]. Risk-focused perinatal screens and assessments with Aboriginal women frequently exacerbate feelings of alienation and disengagement from potentially supportive care [10]. There is an urgent need for culturally safe and effective trauma-aware and healing-informed screening for social, emotional, mental-health and well-being that includes relevant supportive and strength-based follow-up care for Aboriginal mothers.

The Baby Coming You Ready (BCYR) program [10], was co-designed to overcome these barriers and challenges faced by Aboriginal parents during their perinatal care. The BCYR program focuses on a digitized, strengths-based, culturally safe, and holistic perinatal assessment that incorporates all 7 elements of Aboriginal peoples’ social and emotional well-being [11]. The assessment uses iPads with touchscreen images depicting common strengths, worries, and past and present occurrences. Aboriginal voice-overs accompany each slide to guide reflective engagement between the mother and her midwife or health professional. Mothers choose images they relate to while engaging in self-reflection, creating their own personalized story, then prioritizing their strengths and concerns, and designing their own solutions. The assessment automatically generates a clinical event summary, which serves as an individualized follow-up management plan for the mother and health professional. Currently, the BCYR program is operationalized as a model of care in all 6 pilot sites in Western Australia (WA) and effectively replaces all currently required screens for mental-health; family and domestic violence; tobacco; and alcohol and other drugs. While the successful pilot demonstrated increased trust, engagement, honest disclosure, and self-directed management plans, it found that some midwives and managers lacked confidence in conducting culturally considered holistic assessments [10]. Traditional perinatal mental health assessments primarily focus on risk factors, which continue to influence clinical practice and often lead clinicians to overemphasize risk scores and prioritize risk-based discussions during consultations. This reliance may limit trauma-aware and healing-informed care, particularly for Aboriginal mothers [7], highlighting the need for approaches that better support culturally responsive and strengths-based assessments [12].

Over the last decade, advances in digital health and computational technology have driven numerous studies on technology-based approaches to supporting perinatal mental health [13,14]. Among these, artificial intelligence (AI)–based models particularly those using machine learning (ML) and deep learning, have been developed to predict perinatal mental health conditions [15-20]. These models demonstrate the potential to enhance clinical practice by enabling early and accurate detection of depression, facilitating better clinical judgment, and identifying patterns that may be overlooked in manual assessments [21]. Despite these advancements, progress in applying AI to improve health outcomes in Aboriginal populations has been limited [22]. ML models typically trained on data from the general population, often lack cultural relevance and fail to account for unique protective and risk factors, as well as the social determinants of health specific to Aboriginal communities. Moreover, many AI models function as “blackbox” due to their lack of interpretability [23]. However, model transparency is especially critical in health care, particularly for underrepresented populations, where trust and clarity are essential. Explainable artificial intelligence (XAI) has emerged as a promising approach that provides clear explanations for AI and ML algorithm predictions and decision-making processes [24,25]. Such techniques have been successfully applied in various health applications and predictive modeling [26-29].

This study aims to explore different ML techniques to develop a culturally informed, strengths-based AI model for predicting perinatal psychological distress in Aboriginal mothers. The model is built using holistic and culturally contextualized assessment data from the BCYR program. To enhance transparency and clinical relevance, XAI techniques are incorporated to provide clear reasoning behind AI-driven decisions. This approach helps identify, prioritize, and quantify both maternal protective and risk factors, as well as their interactions and impact on perinatal mental health outcomes. By offering deeper insights into Aboriginal perinatal mental health, this model may support more holistic and culturally responsive assessments, ultimately improving clinical decision-making.

## Methods

### Setting and Data Source

The dataset used in this study consists of de-identified data collected from the WA BCYR pilot program [30]. The dataset includes 293 Aboriginal mothers who participated between September 9, 2021, and June 16, 2023, across 6 diverse pilot sites services in metropolitan Perth and regional WA. The BCYR assessment/screen is being offered to all women at pilot sites as part of their routine perinatal care in an additional 30-minute stand-alone appointment [10]. All pregnant women or mothers with infants who accessed participating perinatal services at the pilot sites were eligible to take part in the BCYR assessment. The sampling approach was convenience-based, with the BCYR assessment offered to all eligible Aboriginal mothers attending the pilot sites during the study period.

### Ethical Considerations

Ethics approval for this study was obtained from the Human Research Ethics Committee (HREC) - Western Australia Research Governance Service (RGS000000649), Murdoch University (2021/101), and the Western Australian Aboriginal Health Ethics Committee (WAAHEC; HREC553). Access to deidentified data was granted only to participants who provided informed consent. The information sheet, and a consent button, allowing participants to choose whether or not to participate and share their deidentified data for research purposes, were embedded in the digital application.

### Data Preparation and Preprocessing

Observational units were individual patients, with response variables (psychological distress risk) and predictor variables (demographic, social, and behavioral factors). The skipped question’s answer by the participants was assigned a value of –1.

### Predictors

The original dataset contains 345 variables for each participant, covering a wide range of inquiry domains such as strengths and culturally protective factors, common life events, worries, quality of relationships, childhood experiences, family and domestic violence, and tobacco and alcohol and other drug use. Feature selection was performed using the RF to compute variable importance ranking. The algorithm was configured with 500 trees, and the “mtry” parameter was set to the square root of the total number of variables, rounded down to the nearest integer. Initially, the top 30 most significant variables were selected. These variables were then reviewed by the BCYR research team, which included Aboriginal researchers, both Aboriginal and non-Aboriginal health care professionals, and BCYR digital assessment users. Incorporating their domain knowledge and experience, the final list was narrowed down to 20 predictor variables along with the Kessler-5 item psychological distress scale (K5) output variable, for analysis. The final selected variables are listed in Table 1.

**Table 1:** Selected variables for the prediction model construction.

Code		Question	Variable name	Answer options
**Predictors**				
	fs1.Q225	I feel lonely like I don’t belong or fit in	Feeling Lonely	5: Almost always 4: Often 3: Sometimes 2: A little 1: Hardly ever
	fs1.Q227	I blame myself when things go wrong	Blaming Herself	5: Almost always 4: Often 3: Sometimes 2: A little 1: Hardly ever
	fs1.Q231	Recently I feel like life is not worth living	Life Not Worth Living	1: Never 2: Rarely 3: Sometimes 4: Often 5: Almost always
	fs1.Q214	I feel strong about being a mum	Strong Mum	1: Almost always 2: Often 3: Sometimes 4: A little 5: Hardly ever
	fs1.Q562	How likely is it that you will do your goals?	Goal Likely	1: A lot 2: A fair amount 3: A little bit 4: Not at all
	fs1.Q904	Managing day to day	Managing Day-to-Day	0: Manage well 1: Struggle a bit 2: Struggle a lot
	fs1.Q534	Client agrees to making a plan to keep safe to deal with the safety worries	Keeping Safety Plan	0: Does not agree 1: Client agrees
	fs1.Q455	Are there ever times when gambling bothers you?	Bothered by Gambling	0: Never 1: Sometimes
	fs1.Q228	I make my family proud	Makes Family Proud	1: Almost always 2: Often 3: Sometimes 4: A little 5: Hardly ever
	fs1.Q454	Are there people close to you gambling?	Family Gambles	0: No 1: Sometimes 2: Yes
	fs1.Q664	How many of these children are in your care?	Children in Her Care	0: 0 1: 1 2: 2 3: 3 4: 4 or more
	fs1.Q450	Have you smoked cigarettes?	Smoking Cigarettes in Pregnancy	0: no 1: sometimes 2: yes
	fs1.Q661	Is this your first pregnancy?	First Pregnancy	0: No 1: Yes
	fs1.Q909	Do you have troubles sleeping?	Trouble Sleeping	1: Sleeping well 2: Trouble sleeping (not due to pregnancy/baby)
	fs1.Q922	Secure housing	Need Help with Housing	0: No 1: Yes
	fs1.Q663	How many previous births have you had?	Previous Births	0: 0 1: 1 2: 2 3: 3 4: 4 or more
	fs1.Q71	Are you feeling worried?	Feeling Worried	0: No 1: Yes
	fs1.Q653	Told partner/husband about pregnancy?	Told Partner/Husband	0: No 1: Yes
	fs1.Q204	Is your male partner angry or controlling?	Partner Angry/Controlling	0: No 1: Yes
	fs1.Q195	Is your male partner moody?	Partner Moody	0: No 1: Yes
**Outcome**				
	K5^a^	Psychological distress score category	—^b^	0: low_risk 1: high_risk

^a^K5: Kessler-5 item psychological distress scale.

^b^Not applicable.

### Outcome Variable

The indicator for maternal psychological distress is based on the K5 scale [31]. The K5 scale consists of 5 items, each rated on a 5-point scale from 1 to 5, with all items negatively keyed. The total score ranges from 5 to 25, with a score below 12 indicating low risk (0), and a score of 12 or higher indicating high risk (1) [32]. Five records were excluded from the original dataset due to missing information on the K5 outcome variable. The dataset had a class ratio of 0.65 (low risk) to 0.35 (high risk).

### Model Development and Building

We used 7 ML models to train and evaluate the prediction model on the processed dataset. These models were chosen due to their widespread use and proven effectiveness in health care predictive modeling, particularly with relatively small tabular data [33-35]. The models include random forest (RF) [36], CatBoost (CB) [37], light gradient-boosting machine (LightGBM) [38], extreme gradient boosting (XGBoost) [39], k-nearest neighbor (KNN) [40], and support vector machines (SVM) [41] as blackbox models, and one inherently interpretable glassbox model explainable boosting machines (EBMs) [42]. More descriptions of these selected models are provided in Section I in Multimedia Appendix 1.

### Prediction Performance Evaluation

We used a 10-fold cross-validation strategy for splitting the training and testing data to ensure a fair model training and evaluation process. Hyperparameters for each model were tuned using grid search with cross-validation. Details for hyperparameter tuning were provided in Section III in Multimedia Appendix 1.

Ten-fold was adopted as it is more suited for relatively small datasets, offering a better balance between bias and variances [43]. We then report the average model performance using a comprehensive set of metrics, including accuracy, precision, recall, *F*_1_-score, and area under the curve (AUC). Precision, recall, *F*_1_-score, and AUC are particularly recognized as robust metrics when dealing with imbalanced class ratios. Additionally, 95% CIs are provided for each performance metric to account for uncertainty.

### Model Explanation

Different model explanation techniques were used to investigate how factors influence psychological distress and to compare their outputs. First, EBM, as a glassbox model, is inherently explainable and provides both global explanations for the model’s overall behavior, and local explanations for specific predictions on individual cases [42]. We also applied post-hoc explanation techniques, including Shapley additive explanations (SHAP) [44], local interpretable model-agnostic explanations (LIME) [45,46], and partial dependence plots (PDP) [47], to elucidate the predictions of the best-performing blackbox model. SHAP and LIME can offer both global and local explanations. PDP, as a visualization technique, shows how 1 or 2 selected features impact the predicted outcome while keeping all other features constant. More details on these post-hoc explanation techniques are provided in Section II in Multimedia Appendix 1.

## Results

### Prediction Model Performance

**Figure 1:**
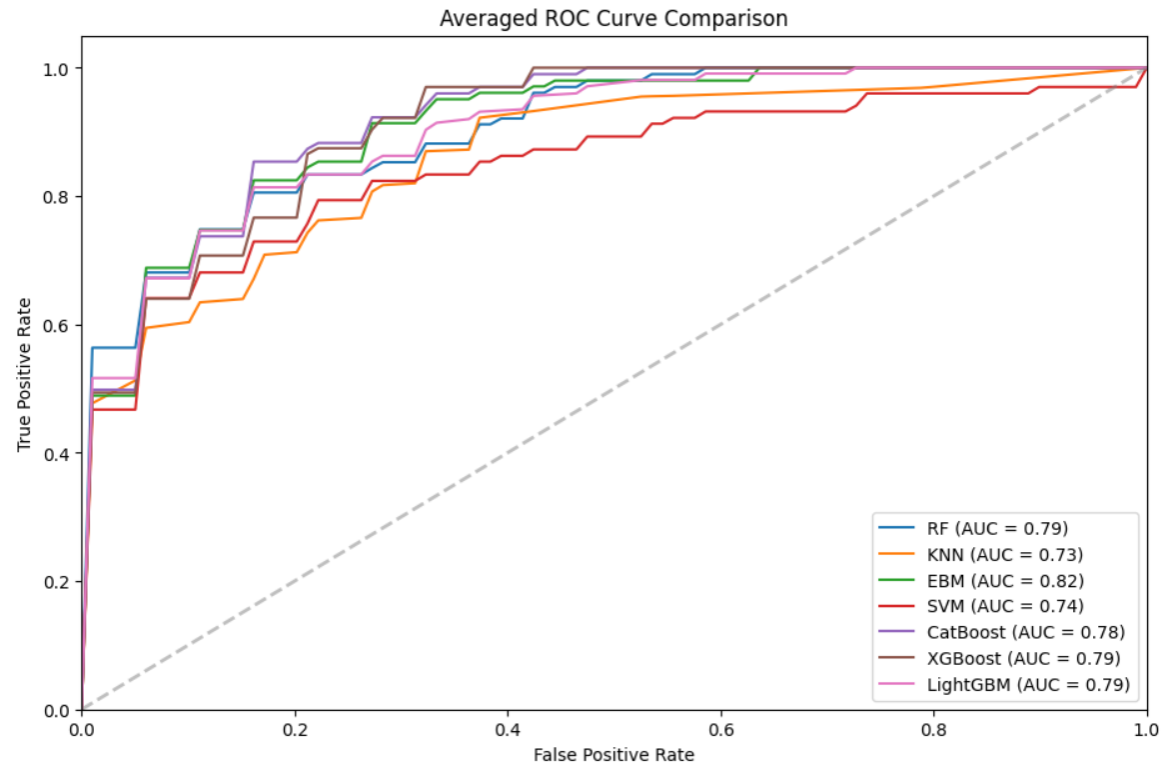
Receiver operating characteristic curve (ROC) plot of all machine learning models. AUC: area under the curve; EBM: explainable boosting machine; KNN: k-nearest neighbor; LightGBM: light gradient-boosting machine; RF: random forest; SVM: support vector machine; XGBoost: extreme gradient boosting.

Table 2 displays the training and evaluation results of all the ML models across various performance metrics, with the best results highlighted in italics. Among black-box models, RF achieved the highest performance, with an accuracy of 0.829, an *F*_1_-score of 0.736, and an AUC of 0.795. The glass-box model EBM outperformed all models, attaining an accuracy of 0.849, an *F*_1_-score of 0.771, and an AUC of 0.821. Ensemble models, including RF, CB, XGBoost, and LightGBM, demonstrated strong predictive performance, all achieving an accuracy above 0.81. KNN and SVM showed slightly lower accuracy (0.798 and 0.794) and comparable AUC values (0.733 and 0.742). In terms of precision and recall, KNN exhibited the highest precision (0.868) but the lowest recall (0.514), leading to a lower *F*_1_-score (0.621). EBM and RF achieved a better balance, with EBM attaining a precision of 0.829, a recall of 0.727, and an *F*_1_-score of 0.771, while RF reached a precision of 0.820, a recall of 0.680, and an *F*_1_-score of 0.736. Figure 1 plots the receiver operating characteristic curve curves of all the models on one set of testing, showing that EBM and RF achieved the highest AUC values.

**Table 2:** Performances of all machine learning models for prediction.

			Accuracy					Precision					Recall					*F*_1_-score					AUC^a^		
			Mean (SD)		95% CI		Mean (SD)			95% CI		Mean (SD)			95% CI		Mean (SD)			95% CI		Mean (SD)			95% CI
**RF^b^**																									
	Training	0.900 (0.05)		0.8700-0.9300		0.914 (0.05)			0.8826-0.9462		0.788 (0.10)			0.7240-0.8513		0.845 (0.08)			0.7961-0.8933		0.874 (0.06)			0.8370-0.9119	
	Testing	0.829 (0.05)		0.7960-0.8617		0.8200 (0.11)			0.7524-0.8869		0.680 (0.11)			0.6099-0.7501		0.736 (0.08)			0.6859-0.7851		0.795 (0.06)			0.7581-0.8318	
**CB^c^**																									
	Training	0.99 (0.02)		0.9778-1.0032		0.997 (0.01)			0.9929-1.0021		0.975 (0.05)			0.9429-1.0077		0.986 (0.03)			0.9664-1.0049		0.987 (0.03)			0.9699-1.0042	
	Testing	0.818 (0.04)		0.7929-0.8439		0.789 (0.07)			0.7430-0.8359		0.669 (0.11)			0.6035-0.7347		0.719 (0.07)			0.6763-0.7620		0.784 (0.05)			0.7522-0.8163	
**XGBoost^d^**																									
	Training	0.933 (0.06)		0.8953-0.9715		0.967 (0.04)			0.9454-0.9894		0.836 (0.15)			0.7405-0.9318		0.891 (0.1)			0.8284-0.9543		0.911 (0.08)			0.8602-0.9625	
	Testing	0.822 (0.04)		0.7954-0.8485		0.807 (0.09)			0.7490-0.8650		0.667 (0.12)			0.5953-0.7392		0.721 (0.08)			0.6710-0.7719		0.786 (0.05)			0.7528-0.8189	
**LightGBM^e^**																									
	Training	0.975 (0.04)		0.9503-1.0002		0.988 (0.03)			0.9719-1.0037		0.94 (0.09)			0.8810-0.9984		0.962 (0.06)			0.9216-1.0015		0.967 (0.05)			0.9346-0.9998	
	Testing	0.822 (0.05)		0.7898-0.8534		0.795 (0.11)			0.7286-0.8604		0.686 (0.14)			0.6003-0.7725		0.726 (0.08)			0.6735-0.7778		0.790 (0.07)			0.7497-0.8305	
**KNN^f^**																									
	Training	1.000 (0.00)		1.000-1.000		1.000 (0.00)			1.000-1.000		1.000 (0.00)			1.000-1.000		1.000 (0.00)			1.000-1.000		1.000 (0.00)			1.000-1.000	
	Testing	0.798 (0.07)		0.7524-0.8430		0.868 (0.13)			0.7860-0.9491		0.514 (0.21)			0.3846-0.6426		0.621 (0.16)			0.5204-0.7216		0.733 (0.10)			0.6709-0.7948	
**EBM^g^**																									
	Training	0.886 (0.01)		0.8797-0.8927		0.899 (0.02)			0.8882-0.9091		0.764 (0.02)			0.7505-0.7770		0.826 (0.02)			0.8152-0.8359		0.858 (0.01)			0.8506-0.8661	
	Testing	0.849 (0.05)		0.8170-0.8814		0.829 (0.10)			0.7689-0.8900		0.727 (0.11)			0.6599-0.7946		0.771 (0.09)			0.7169-0.8245		0.821 (0.06)			0.7829-0.8593	
**SVM^h^**																									
	Training	0.858 (0.05)		0.8275-0.8894		0.920 (0.04)			0.8943-0.9466		0.652 (0.12)			0.5804-0.7236		0.760 (0.09)			0.7055-0.8150		0.812 (0.06)			0.7715-0.8517	
	Testing	0.794 (0.07)		0.7514-0.8373		0.805 (0.14)			0.7195-0.8906		0.570 (0.18)			0.4604-0.6796		0.650 (0.13)			0.5675-0.7335		0.742 (0.09)			0.6880-0.7966	

^a^AUC: area under the curve.

^b^RF: random forest.

^c^CB: CatBoost.

^d^XGBoost: extreme gradient boosting.

^e^LightGBM: light gradient-boosting machine.

^f^KNN: k-nearest neighbor.

^g^EBM: explainable boosting machine.

^h^SVM: support vector machines.

### Explanation Results

#### Global Interpretation by EBM, SHAP, and RF

**Figure 2:**
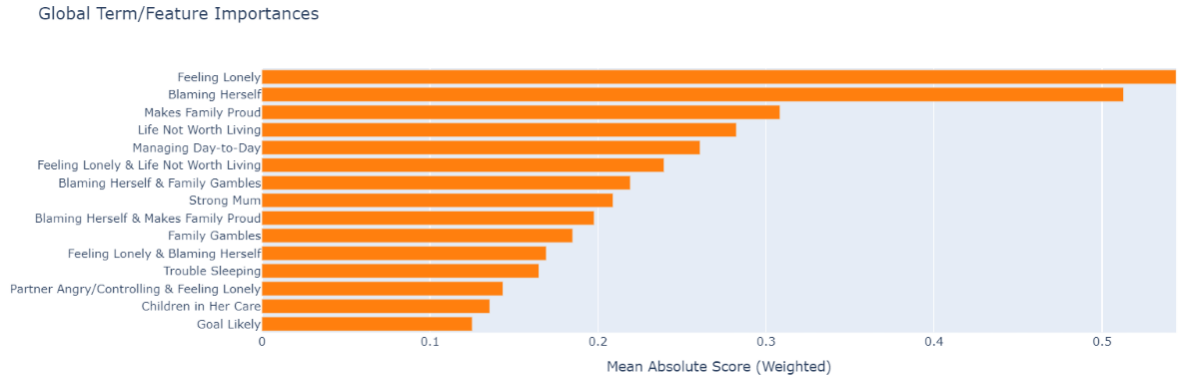
Global feature importance interpretation from glassbox model explainable boosting machine (EBM).

As EBM demonstrates high predictive performance and operates as a transparent glassbox model, we generated the explanation from EBM and illustrated the global feature importance over the whole dataset in Figure 2. Longer bars in the figure indicate the higher importance of features in the model’s predictions. It is noteworthy that specific features exert a more significant influence on the decision-making process of the EBM model. For instance, the feature “Feeling Lonely” emerges as the most impactful, suggesting that a participant’s response to a reflection concerning feelings of loneliness, might serve as a robust predictor for perinatal mental health risk. This is followed by “Blaming Herself” and “Makes Family Proud,” indicating that these 2 features have a higher contribution to the overall prediction (risk or protective). Other features, such as “Managing Day-to-Day” and “Strong Mum,” also demonstrate a notable impact in relation to the K5 target outcome.

**Figure 3:**
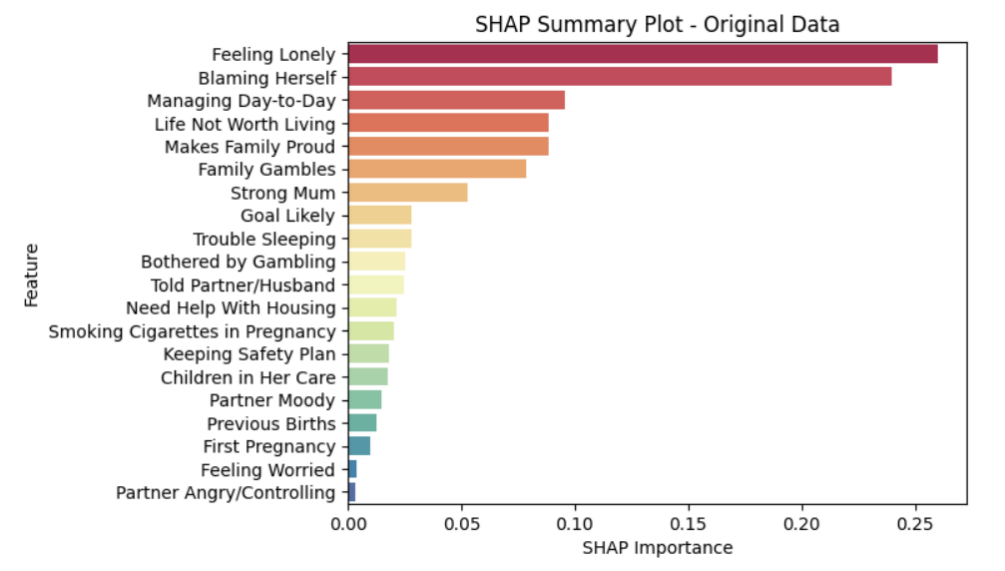
Global feature importance interpretation from post-hoc Shapley additive explanations (SHAP).

**Figure 4:**
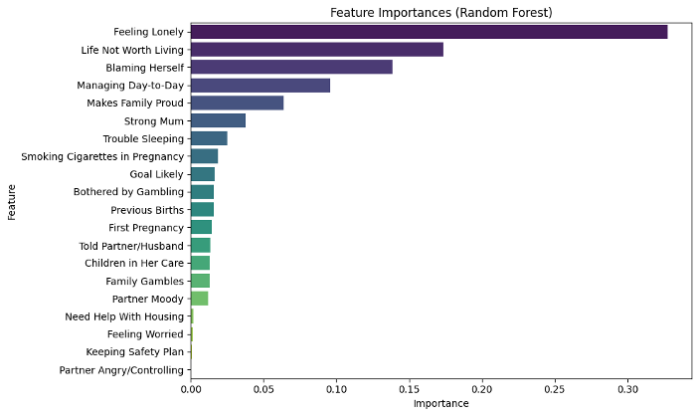
Feature importance ranking from random forest (RF).

For the high-performing black-box RF model, we used SHAP to also gain insights into the model’s explanation. We displayed the global feature importance ranking in Figure 3. The most important features, including “Feeling Lonely,” “Blaming Herself,” “Managing Day-to-Day,” “Life Not Worth Living,” and “Makes Family Proud,” exactly overlap with the top features selected from EBM. Additionally, features related to questions concerning “Family Gambles,” “Strong Mum,” “Goal Likely,” and “Trouble Sleeping” were subsequently ranked, aligning with EBM’s ranking tier as well. We also provided the RF feature importance rankings in Figure 4. The top-ranked features including “Feeling Lonely,” “Life Not Worth Living,” “Blaming Herself,” “Managing Day-to-Day,” “Strong Mum,” and “Trouble Sleeping” are largely consistent with the key features identified by SHAP and EBM. There are some variations in ranking. For example, RF assigns higher importance to “Life Not Worth Living” and “Managing Day-to-Day,” while SHAP emphasizes “Family Gambles” and LIME highlights interaction terms such as “Feeling Lonely” & “Life Not Worth Living.”

#### Local Interpretation by EBM, SHAP, and LIME

**Figure 5:**
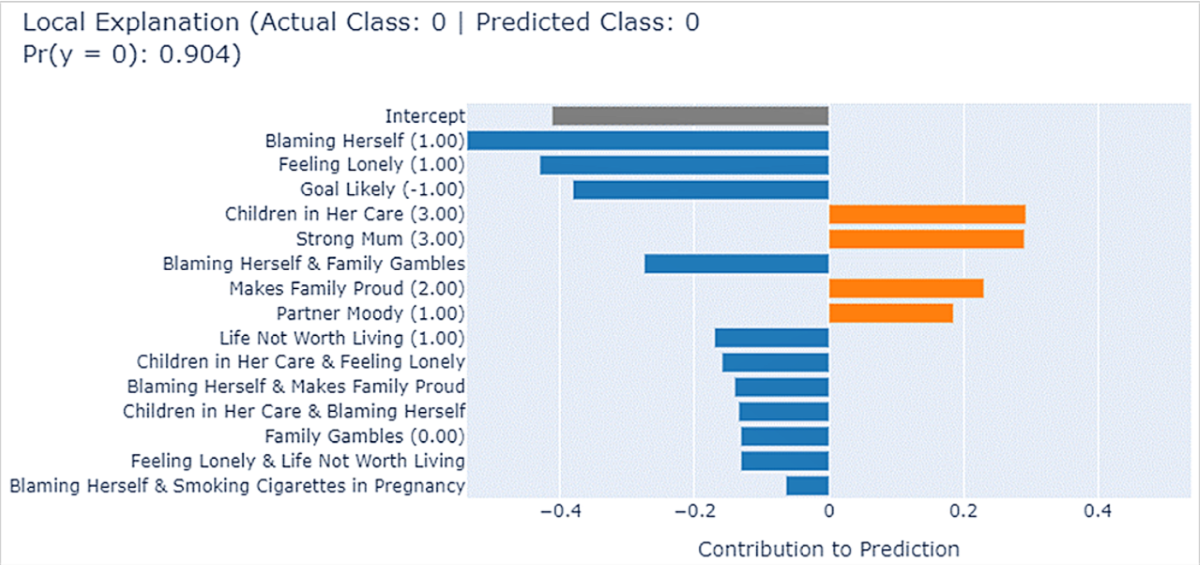
Local explanation for Instance I by explainable boosting machine (EBM).

EBM can also provide an interpretation of the model’s prediction on an individual instance. Figure 5 shows one Aboriginal woman who is low risk, labeled “Instance I,” it shows the specific contributions of each feature and their interactions in combination toward risk prediction for this individual by EBM. EBM accurately predicts this individual as “low risk” (class=0), with a high probability score of 0.904. The y-axes of the plot show impactful features and their corresponding values in brackets. Specifically, contributing features highlighted in blue, such as “Blaming Herself”=1.00 (hardly ever) and “Feeling Lonely”=1.00 (hardly ever), contribute to shifting the model’s prediction toward the low-risk category. These 2 features stand out, suggesting that “hardly ever feeling lonely” and “hardly ever blaming herself” are strongly protective factors for this Aboriginal mother. In addition, the combination of “Blaming Herself”=1.00 (hardly ever) and “Family Gambles”=0.00 (loved ones do not gamble) shows an additive positive influence, pushing the prediction toward low risk. Conversely, contributing features displayed in orange in the figure, would have pushed the model’s prediction away from low risk. For example, “Children in Her Care”=3 (having 3 children in care) and “Strong Mum”=3 (sometimes), and “Makes Family Proud”=2 (often) and “Partner Moody”=1 (yes), are risk factors that increase this woman’s risk. In our analysis, we noted that “Makes Family Proud” and “Strong Mum” were highly protective only when women selected the top rating: “Always.”

**Figure 6:**
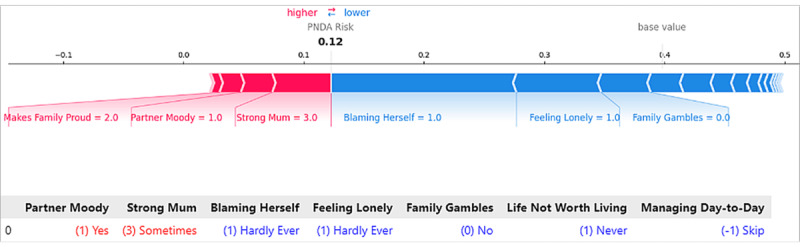
Local explanation for Instance I by Shapley additive explanations (SHAP). PNDA: perinatal depression.

Using SHAP, we generated force plots in Figure 6 to visualize the contributions of important features to the prediction for Instance I. The key features influencing the prediction for this individual are depicted in red and blue. Red indicates features that elevated the model’s score toward high risk. Blue signifies features that reduce the risk. Features having a greater impact on prediction scores are located closer to the dividing boundary between red and blue in the figure. Therefore, the strongly protective factors contributing to this low-risk prediction, including “hardly ever blames herself,” “hardly ever feels lonely,” and “close loved ones do not gamble,” align with the EBM’s individual interpretation. Additionally, “sometimes feels strong about being a mum” and “moody partner” tended to push the prediction toward higher risk, which again is similar to EBM’s interpretation.

**Figure 7:**
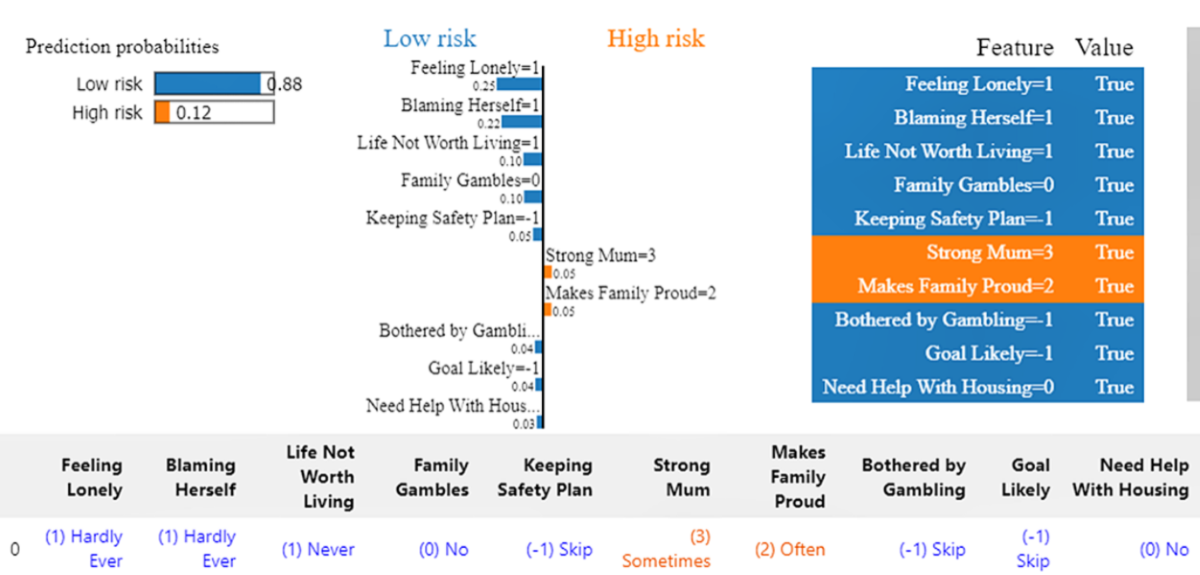
Local explanation for Instance I by local interpretable model-agnostic explanations (LIME).

We used LIME to further explore individual influencing features for the same woman. Overall, the results were very similar to EBM and SHAP technologies. Figure 7 illustrates that the most influential features in predicting low risk were “hardly ever feels lonely,” “hardly ever blames herself,” “never feels life is not worth living,” and “close loved ones do not gamble.” Interestingly LIME uniquely identified that “needs no help with housing” was a contributor to low risk, which was not highlighted by EBM or SHAP.

**Figure 8:**
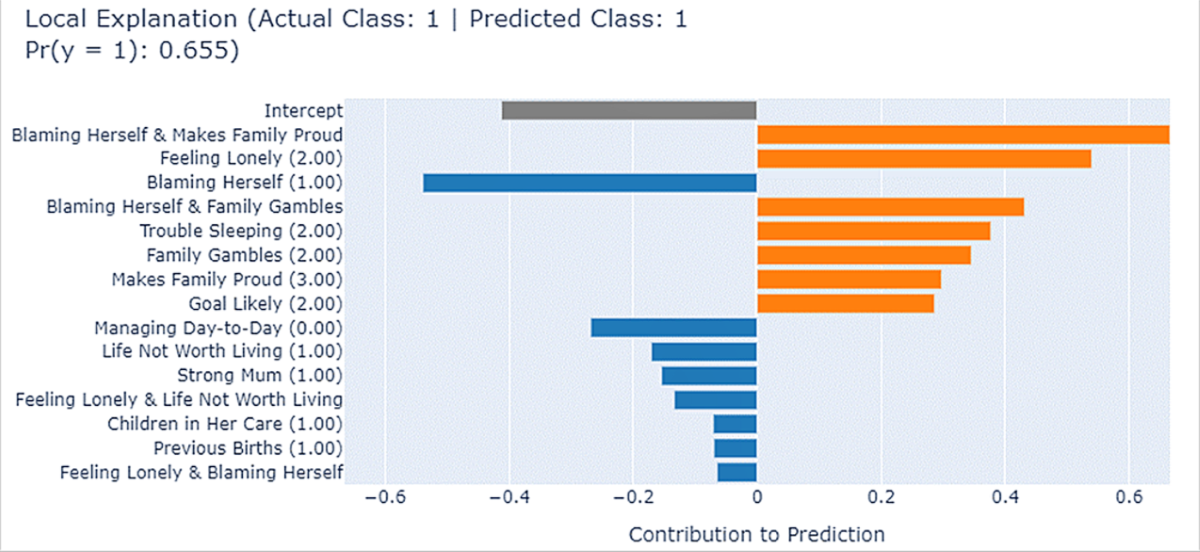
Local explanation for Instance II by explainable boosting machine (EBM).

Figure 8 provides the EBM’s local explanation for another Aboriginal woman labeled “Instance II,” who was identified as being at higher risk. EBM accurately predicted the outcome with a probability score of 0.655. Several protective factors highlighted in blue contributed to mitigating the risk include “Blaming Herself”=1 (hardly ever blames herself), “Managing Day-to-Day”=0 (manages day-to-day well), “Life Not Worth Living”=1 (never feels life is not worth living), “Strong Mum”=1 (almost always feels strong about being a mum), “Children in Her Care”=1 (having one child in care), and “Previous Births”=1 (one previous birth). Several factors in red significantly influencing the prediction decision toward high risk include “Feeling Lonely”=2 (feels a little lonely), “Trouble Sleeping”=2 (having trouble sleeping), “Family Gambles”=2 (close loved ones gamble), “Makes Family Proud”=3 (sometimes makes her family proud), and “Goal Likely”=2 (a fair amount). While the protective features provided some mitigating effects, the stronger influence of risk factors ultimately influenced the model’s high-risk prediction.

**Figure 9:**
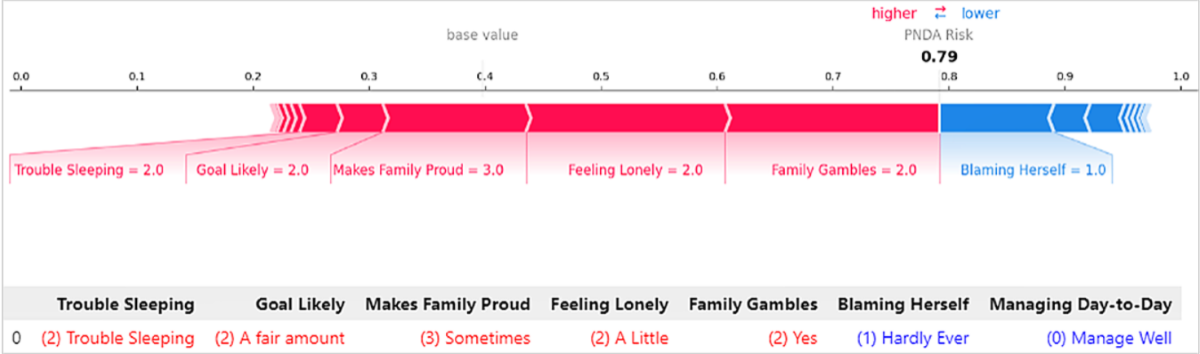
Local explanation for Instance II by Shapley additive explanations (SHAP). PNDA: perinatal depression.

Figure 9 shows the prediction interpretation for the same individual using SHAP. A consistent group of answers to the questions that drive the prediction toward a high risk was identified, including “Family Gambles,” “Feeling Lonely,” “Makes Family Proud,” “Goal Likely,” and “Trouble Sleeping,” although the significance level ranking shows slight differences. Meanwhile, not “Blaming Herself” and “Managing Day-to-Day” were identified as mitigating factors that attempt to reduce this high-risk prediction.

**Figure 10:**
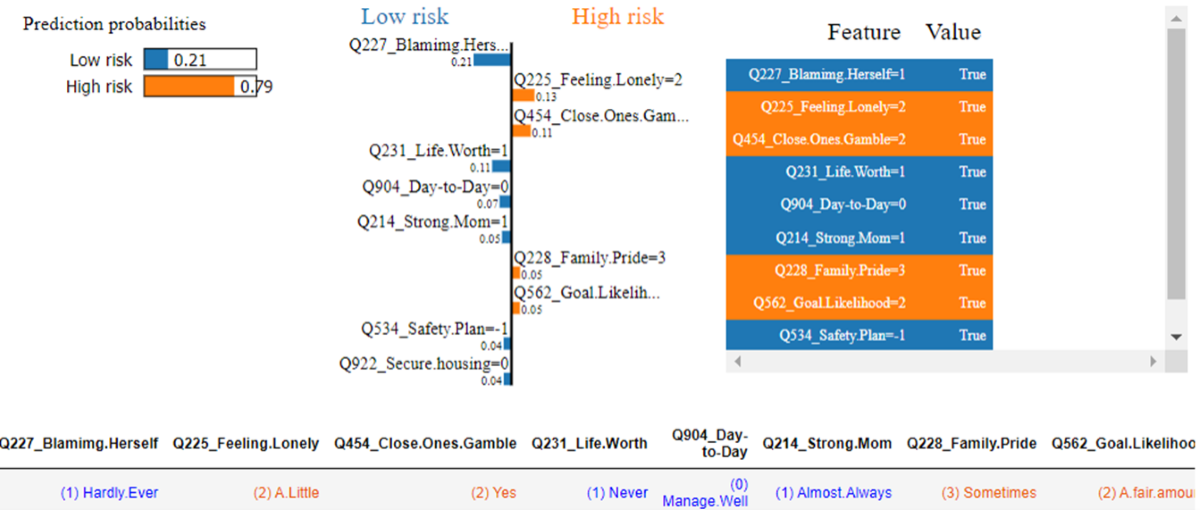
Local explanation for Instance II by local interpretable model-agnostic explanations (LIME).

Figure 10 provides LIME’s local interpretation of the same individual. The protective factors largely align with the other 2 methods, except for “Need Help with Housing”=0, chosen as a protective factor by LIME, which was not picked as a top protective factor by the other 2 methods. LIME selected the same group of risk factors as SHAP, except for “Trouble Sleeping,” which was not chosen by LIME but was selected by both SHAP and EBM.

#### High Influential Factor Interpretation by PDP

**Figure 11:**
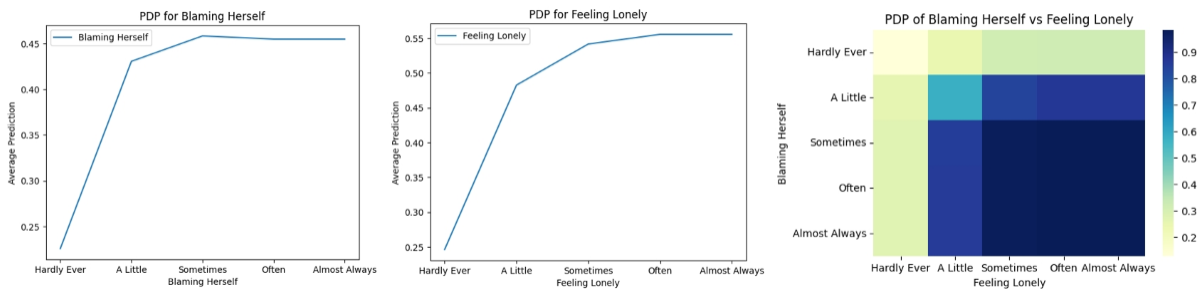
Partial dependence plots (PDP) for Blaming Herself; PDP for Feeling Lonely; PDP for Blaming Herself versus Feeling Lonely.

Through interpretation conducted, we identified 2 highly significant questions impacting the prediction outcomes: “Blaming Herself” and “Feeling Lonely”. We further adopted their corresponding PDP plots to reveal their individual relationships with the average predicted outcome shown in Figure 11. In the case of “Blaming Herself,” mothers who hardly ever blame themselves are associated with a low risk. However, starting from “A Little” and progressing to “Sometimes,” “Often,” and “Almost Always,” there is a significant increase in the predicted risk of perinatal mental health issues. This suggests that as the frequency of self-blame rises, even starting from “A Little,” the associated risk shows a significant increasing trend. There is little difference in the impact between the categories “Sometimes,” “Often,” and “Almost Always,” as they all show a significant level of relation with higher risk. Similar observations were made for the case of “Feeling Lonely,” where mothers who reported “Hardly Ever” feeling lonely are associated with a low risk.

We further generated a PDP plot to visualize the interplay between these 2 significant factors. The heatmap color gradient represents the predicted risk level, with light cream indicating a low risk (closer to 0) and purple representing a high risk (closer to 1). Mothers who reported “Hardly Ever” blaming themselves and “Hardly Ever” feeling lonely are in the lightest zone, suggesting the lowest predicted risk. However, the deeper color zone is pronounced for respondents who reported feeling lonely starting from “A Little” and beyond and blaming themselves starting from “A Little” and beyond. This combined emotional state of frequent loneliness and self-blaming puts them at a much higher predicted risk for perinatal mental health issues. Regardless of whether the response was “Sometimes,” “Often,” or “Always” for loneliness and self-blame, it led to the highest level of risk, with almost the same effect.

## Discussion

### Principal Findings

The prediction model performance analysis indicates that EBM and RF performed best overall, offering a strong balance between accuracy, *F*_1_-score, and AUC. Ensemble models (RF, CB, XGBoost, and LightGBM) demonstrated strong predictive performance, benefiting from their ensemble-based architecture, which enhances generalization and robustness. While CB, XGBoost, and LightGBM are powerful, they may be more prone to overfitting on small datasets. In contrast, RF uses bagging, which reduces variance and tends to be more robust for small datasets. Among the models, KNN exhibited high precision but low recall, indicating a tendency to miss high-risk cases, which could be a critical limitation for mental health risk detection. In contrast, EBM and RF provided a better balance, making them more suitable for this task.

The global feature importance analysis using EBM, SHAP, and RF identified key predictors of perinatal mental health risk and revealed important interactions between features. The question concerning “Feeling Lonely” consistently emerged as the most influential predictor across models, followed by questions concerning “Blaming Herself,” “Makes Family Proud,” “Life Not Worth Living,” and “Managing Day-to-Day.” Moreover, EBM showed specific multidimensional interactions that add increased weighting to the model’s predictions. For example, “Feeling Lonely” in combination with “Life Not Worth Living,” “Blaming Herself,” or “Partner Angry/Controlling” placed greater predictive power. Similarly, interactions between “Blaming Herself” and “Family Gambles” or “Makes Family Proud” were identified as key joint effects affecting model predictions.

Variations in feature rankings across EBM, SHAP, and RF likely arise from methodological differences in how global feature importance is measured. RF determines importance based on node splits and impurity reduction but does not explicitly capture feature interactions. SHAP estimates feature contributions by considering their marginal effects and interactions across all predictions. EBM computes global importance by aggregating the learned effects of each feature in an additive framework while explicitly modeling interactions through pairwise terms. By using these different global feature importance methods, we can get a more comprehensive understanding of feature importance and uncover key patterns that enhance model interpretability.

### Comparison to Prior Work

Recent advancements in AI-driven predictive models for perinatal mental health have demonstrated varying levels of effectiveness. Previous studies introduced an EBM trained with Aboriginal lived experiences, highlighting the need for culturally sensitive AI applications [27]. Similarly, another study showed that ML models, particularly RF and SVM, could effectively predict psychological distress among Aboriginal perinatal mothers [32]. Other studies have explored the broader application of AI in perinatal health, such as AI’s role in predicting preterm birth and postpartum depression [20,48]. Our study builds upon these findings by integrating XAI techniques and incorporating Aboriginal knowledge and lived experiences to improve transparency in decision-making and support the development of culturally safe AI applications in perinatal mental health.

At the individual level, where responses are highly personal, local explanations from XAI techniques provided case-specific insights, distinguishing between protective and risk factors and illustrating their respective contributions. In combination with global feature importance results, positive family relationships emerged as a key protective factor in mitigating poor perinatal mental health, aligning with findings from Ratajczak [9] and Carlin et al [12]. Similarly, risk factors such as feelings of loneliness and poor partner relationships were consistent with the findings of Carlin et al [12]. This study may offer new ways to identify protective and risk factors in Aboriginal perinatal mental health from an explainable AI-based quantitative perspective and predictive modeling approach. Such models could facilitate the early detection of at-risk individuals and support more personalized, culturally sensitive, strengths-based care.

### Limitations and Future Directions

This study has several limitations that should be acknowledged. First, our model was trained on the dataset obtained through convenience sampling, without a formal sample size calculation. The limited sample size and nonrandom sampling approach may introduce selection bias, potentially limiting the model’s generalizability and increasing the risk of overfitting. While cross-validation techniques were applied to mitigate these risks and assess generalization capability, they cannot fully compensate for the limitations posed by the sampling method and dataset size. Second, the absence of established population parameters prevents direct statistical comparisons with broader populations, making it challenging to assess selection bias and affecting the study’s generalizability. Third, potential biases in assessment responses, such as nonresponse and social desirability bias, may affect data quality and influence model outputs. While XAI techniques provide a way to identify potential distortions, they do not fully quantify these biases, making it difficult to comprehensively assess model fairness and accuracy. Future research should focus on expanding the dataset by incorporating a more diverse and representative sample across different regions, performing external validation using data from different regions, and systematically assessing model fairness. These steps could help enhance the model’s performance, generalizability, and reliability in practice. Fourth, there is a slight imbalance in the outcome class ratio between low risk and high risk, at 0.65 versus 0.35. Given that the class ratio is relatively moderate and ML models especially ensemble techniques like RF can naturally handle this level of imbalance, and class imbalance-robust performance metrics were used for evaluation, no class imbalance techniques were applied. In the future, as the dataset grows and if the class imbalance increases, additional techniques could be implemented to further improve predictive performance. Fifth, the current visual outputs generated by the XAI techniques can be refined through the co-design process to improve their readability and explainability for Aboriginal women and clinicians. Creating a user-friendly, culturally sensitive visual prediction model will ensure that all practitioners can accurately and responsively interpret the results in practice.

### Conclusions

We developed and evaluated several ML models powered by XAI techniques to predict perinatal psychological distress in Aboriginal mothers. The explanations provided by different XAI techniques revealed largely consistent patterns of influential protective and risk factors, their interactions, and their impact on prediction outcomes. Continuous collaboration informed by Aboriginal knowledge and lived experience, will further enhance the model. Such a model may have the potential to assist health care professionals in providing more culturally sensitive clinical reasoning, improving holistic assessment interpretations, and reducing unnecessary child protection notifications. Future studies are needed for clinical validation.

## Data Availability

The datasets analyzed during this study are not publicly available due to data governance considerations. They may be available from the corresponding author on reasonable request.

## Appendix

Multimedia Appendix 1 Detailed machine learning prediction models, post hoc explanation techniques, and hyperparameter tuning.

## Abbreviations

AI: artificial intelligence
AUC: area under the curve
BCYR: Baby Coming You Ready
CB: CatBoost
EBM: explainable boosting machine
HREC: Human Research Ethics Committee
K5: Kessler-5 item psychological distress scale
KNN: k-nearest neighbor
LightGBM: light gradient-boosting machine
LIME: local interpretable model-agnostic explanations
ML: machine learning
PDP: partial dependence plots
PNDA: perinatal depression and anxiety
RF: random forest
SHAP: Shapley additive explanations
SVM: support vector machine
WA: Western Australia
WAAHEC: Western Australian Aboriginal Health Ethics Committee
XAI: explainable artificial intelligence
XGBoost: extreme gradient boosting
