# Supplementary material for: Investigating Protective and Risk Factors and Predictive Insights for Aboriginal Perinatal Mental Health: Explainable Artificial Intelligence Approach
Source: J Med Internet Res. 2025 Apr 30;27:e68030. doi: 10.2196/68030 (PMC12079063; doi:10.2196/68030)
Supplement: Multimedia Appendix 1 [file jmir_v27i1e68030_app1.docx]

# *Multimedia Appendix 1*

# Machine Learning Prediction Model

Based on the 'no free lunch' theorem, we explored several popular machine learning models on the processed dataset and evaluated their performance. Specifically, we employed both traditional blackbox models and a glassbox model to assess and compare their predictive performance. Notably, the glassbox model offers inherent interpretability, while black-box models are non-interpretable but the interpretability of can be achieved through post-hoc agnostic explanation techniques.

*Traditional ML models*

Six traditional machine learning algorithms—the models include Random Forest (RF) [36], CatBoost (CB) [37], Light Gradient-Boosting Machine (LightGBM) [38], eXtreme Gradient Boosting (XGBoost) [39], K-Nearest Neighbor (KNN) [40] and Support Vector Machines (SVM) [41]— were selected for training and evaluating the prediction model. These choices were made based on their widespread use and proven effectiveness in mental health research, particularly when dealing with tabular data from relatively small datasets [33-35]. RF is a classic ensemble learning technique that builds multiple decision trees during training and combines their decisions to make predictions [36]. This process, while effective, is often seen as opaque because it integrates knowledge learned from multiple individual weak classifiers, making it a black-box model. CatBoost, LightGBM, and XGBoost are also ensemble learning methods. CatBoost is naturally capable of handling categorical features without preprocessing and employs a novel schema for calculating leaf values during tree structure selection, which helps reduce overfitting [37]. LightGBM incorporates Gradient-Based One-Side Sampling (GOSS) and Exclusive Feature Bundling (EFB) to accelerate training while maintaining high accuracy [38]. XGBoost features in its scalability in distributed or memory-limited settings, integrating several key system and algorithmic optimizations [39]. These include a specialized tree learning algorithm for handling sparse data, a theoretically justified weighted quantile sketch procedure for efficient proposal calculation, and a cache-aware block structure for out-of-core tree learning. SVM, another widely used algorithm, aims to find the optimal hyperplane that separates different classes with a maximal margin. Its effectiveness lies in its ability to classify complex, high-dimensional, and non-linear relationships within the data [41]. SVM is generally considered non-interpretable, especially for high-dimensional data. KNN, in contrast, is an instance-based learning algorithm that classifies a data point based on the classifications of its neighbors [40]. While KNN may offer some interpretability, considering that it can explain predictions based on the proximity of similar instances using features, this reliance on proximity can be heavily influenced by parameter choices, such as the value of K. Moreover, in high-dimensional spaces, the curse of dimensionality may cause nearest neighbors to lose representativeness, diminishing the effectiveness of proximity-based explanations. Also, KNN's explainability could also fall short of providing a clear understanding of the underlying relationships within the data, which is often crucial information for clinical research. Therefore, there remains a need for further explanation techniques to enhance its interpretability.

## Glassbox ML model

In addition to the machine learning models mentioned above, we employed Explainable Boosting Machines (EBM) for the prediction model. Unlike the other models, EBM is a glassbox model that is inherently explainable for its predictions based on feature importance. This makes it easier to interpret and understand the model's decisions. Moreover, EBM can offer explanations of both the overall model behavior and individual predictions [42]. Specifically, EBM is a tree-based, gradient boosting Generalized Additive Model (GAM) with automatic pairwise interaction identification. The contribution of each feature to the final prediction can be visualized and sorted, providing an explanation. The final EBM model is a sum of one-dimensional models for each feature and two-dimensional models for potential pairwise interaction terms. This sum is then passed through a logistic function in the case of classification tasks to ensure the output lies between 0 and 1, representing probabilities of the positive class. In general, EBM can be represented by the following form:

| $g(E[y]) = \beta_{0}+\sum f_{i}(x_{i})+\sum f_{ij}(x_{i},x_{j})$ | (1) |
| --- | --- |

where $g(\cdot)$ is link function, which transforms the expected value of the target variable $y$ depending on the classification or regression problem. $\beta_{0}$ is the intercept, representing the expected value of the prediction outcome when all the features are equal to zero. $f_{i}\left( x_{i} \right)$is a learned function based on tree-based gradient boosting for each individual feature $x_{i}$, indicating its contribution to the prediction outcome. $f_{ij}(x_{i},x_{j})$ is another learned function based on tree-based methods for a pair of features $(x_{i},x_{j})$, modelling their features’ interactions on the prediction [42]. To make individual prediction, each function $f_{i}$ and $f_{ij}$ act as a lookup table per feature and two-dimensional features, respectively, returning a term contribution. These term contributions are then simply added up and passed through the link function $g(\cdot)$ for the final prediction.

# Post-hoc Explanation Techniques

We employed several typical post-hoc agnostic explanation techniques to elucidate the black-box model’s predictions, which are briefly reviewed below.

## SHAP (SHapley Additive exPlanations)

SHAP is a model-agnostic technique designed to elucidate the outputs of black-box ML models at both individual and global levels. Rooted in cooperative game theory, SHAP computes Shapley values to determine a fair distribution of the prediction among the features of a given data instance. The Shapley value explanation is represented as an additive feature attribution method using a linear model. The SHAP model can be represented as follows [44]:

| $g\left( z^{'} \right) = \phi_{0}+\sum_{j = 1}^{M} \phi_{j}z_{j}^{'}$ | (2) |
| --- | --- |

where $g$ is the model, $z^{'}\in\left\{ 0, 1 \right\}^{M}$ is the coalition vector, $M$ is the maximum coalition size, and $\phi_{j}\mathcal{\in R}$ is the feature attribution for feature $j$, representing Shapley values. The larger the absolute Shapley values, the more important the associated features. Furthermore, Shapley values can be aggregated into global explanations. After running SHAP for every instance, a matrix of Shapley values, with one vector row per data instance and one column vector per feature, can be formulated. Analyzing this Shapley value matrix enables us to interpret the entire model at the global level. Kernel SHAP is a specific technique for approximating Shapley values through a sampling process, with the sampled instances weighted using the SHAP kernel based on the coalition’s weight in the Shapley value estimation.

## LIME (Local Interpretable Model-Agnostic Explanations)

LIME is a widely adopted model-agnostic technique designed to elucidate individual predictions by constructing surrogate local models that approximate the output of a black-box model, thus offering the capacity for local interpretability [45, 46]. This technique tests how the machine learning model's predictions change in response to variations in the input data. Specifically, LIME generates a perturbed dataset based on the individual instance of interest and its corresponding predictions from the black-box model. Subsequently, LIME utilizes this perturbed dataset with proximity weightings to train an interpretable model to approximate the black-box model's decision-making process. The LIME local surrogate model optimization problem can be represented as follows:

| $\min_{g\in G} L \left( f, g, \pi_{x} \right)+\Omega(g)$ | (3) |
| --- | --- |

The explanation model $g$ for the individual instance $x$ of interest can be a simple interpretable model, e.g., linear regression or a decision tree, that minimizes the square loss function $L$, which measures how close the prediction of the explanation model is to the prediction of the original black-box model $f$. $\pi_{x}$ is the proximity measure deciding the perturbed instance neighbourhood around instance $x$ that are used for the explanation. $\Omega(g)$ is the regularization term to control the model complexity.

## PDP (Partial Dependence Plots)

PDP is a global explanatory, model-agnostic visualization technique that displays the marginal effect of one or two features on the predicted outcome of a black-box ML model while holding all other features constant [47]. This technique allows for an intuitive understanding of the relationship between the input features and the model's predictions, especially for non-technical background stakeholders seeking insights into the model's behavior. Specifically, first, users choose a feature of interest. Once a range of values for the chosen feature is established, based on the user's interest in observing partial dependence, the model computes predictions for each value within that specified range. Simultaneously, all other features are held constant, either fixing them at specific values or by using their average values. Finally, the predicted outcomes against the values of the selected feature are plotted, resulting in a graphical representation of how changes in that specific feature influence the model’s predictions.

# Hyperparameter tuning

The model training was conducted using Python, and hyperparameter tuning was performed for the selected machine learning models to optimize their performance. We utilized RandomizedSearchCV from the Scikit-Learn library, which enables an efficient search over predefined hyperparameter spaces by randomly sampling combinations from specified ranges. Each RandomizedSearchCV run was configured with 50 iterations (n_iter = 50) and evaluated using 10-fold stratified cross-validation to ensure robustness. The best-performing hyperparameters were selected based on accuracy. RF, SVM, and KNN models were implemented using Scikit-Learn, while XGBoost, LightGBM, and CatBoost (CB) were implemented using their respective packages. The final hyperparameter configurations are summarized in Table S1.

**Table S1**. Hyperparameter configurations.

| Hyperparameter | Search Range / Values | Selected Value |
| --- | --- | --- |
| RF | | |
| max_depth | [5, 10, 15, 20, 25, 26, 27, 28, 29, 30] | 29 |
| n_estimators | [20, 25, 30, 35, 40, 45, 50, 100] | 45 |
| max_features | [1, 2, 3, 4, 5, 6, 7, 8, 9] | 7 |
| min_samples_leaf | np.arange(2, 15) (i.e., 2 through 14) | 4 |
| criterion | ['gini', 'entropy'] | gini |
| bootstrap | [True, False] | False |
| CB | | |
| iterations | [100, 200, 500] | 500 |
| depth | [4, 6, 8] | 8 |
| learning_rate | [0.01, 0.05, 0.1] | 0.05 |
| l2_leaf_reg | [1, 3, 5, 7, 9] | 3 |
| XGBoost | | |
| n_estimators | [100, 200, 300] | 200 |
| max_depth | [3, 5, 7, 10] | 7 |
| learning_rate | [0.01, 0.05, 0.1] | 0.05 |
| subsample | [0.5, 0.7, 1.0] | 1.0 |
| colsample_bytree | [0.5, 0.7, 1.0] | 0.5 |
| gamma | [0, 1, 5] | 0 |
| LGBM | | |
| n_estimators | [100, 200, 300] | 300 |
| min_child_samples | [1, 2, 3, 5, 10, 20] | 3 |
| max_depth | [-1, 5, 7, 10] | -1 |
| learning_rate | [0.01, 0.05, 0.1] | 0.01 |
| num_leaves | [2, 3, 4, 5, 10, 15, 20, 31] | 10 |
| subsample | [0.5, 0.7, 1.0] | 1.0 |
| colsample_bytree | [0.5, 0.7, 1.0] | 0.7 |
| KNN | | |
| n_neighbors | range(1, 26) (i.e., 1 through 25) | 8 |
| weights | ['uniform', 'distance'] | distance |
| algorithm | ['auto', 'ball_tree', 'kd_tree', 'brute'] | kd_tree |
| EBM | | |
| learning_rate | [0.01, 0.1, 0.2, 0.5, 0.8, 1] | 0.01 |
| max_leaves | [3, 5, 10] | 3 |
| max_rounds | [50, 100] | 50 |
| SVM | | |
| C | [0.1, 1, 10, 100] | 10 |
| gamma | [1, 0.1, 0.01, 0.001] | 0.001 |
| kernel | ['linear', 'rbf', 'poly', 'sigmoid'] | rbf |
| degree | [2, 3, 4] | 3 |
| shrinking | [True, False] | True |
| probability | [True] | True |
